# Combined Effects of Rosuvastatin and Exercise on Gene Expression of Key Molecules Involved in Cholesterol Metabolism in Ovariectomized Rats

**DOI:** 10.1371/journal.pone.0159550

**Published:** 2016-07-21

**Authors:** Emilienne Tudor Ngo Sock, Gaétan Mayer, Jean-Marc Lavoie

**Affiliations:** 1 Department of kinesiology, Université de Montréal, Montréal (QC) Canada; 2 Laboratory of molecular cell biology, Montreal heart institute, Montréal (QC) Canada; 3 Department of pharmacology, Faculté de médecine, Université de Montreal, Montréal (QC) Canada; 4 Department of medicine, Faculté de médecine, Université de Montréal, Montréal (QC) Canada; 5 Faculté de pharmacie, Université de Montréal, Montréal (QC) Canada; University of Insubria, ITALY

## Abstract

The purpose of this study was to investigate the effects of three weeks of rosuvastatin (Ros) treatment alone and in combination with voluntary training (Tr) on expression of genes involved in cholesterol metabolism (LDLR, PCSK9, LRP-1, SREBP-2, IDOL, ACAT-2 and HMGCR) in the liver of eight week-old ovariectomized (Ovx) rats. Sprague Dawley rats were Ovx or sham-operated (Sham) and kept sedentary for 8 weeks under a standard diet. Thereafter, rats were transferred for three weeks in running wheel cages for Tr or kept sedentary (Sed) with or without Ros treatment (5mg/kg/day). Six groups were formed: Sham-Sed treated with saline (Sal) or Ros (Sham-Sed-Sal; Sham-Sed-Ros), Ovx-Sed treated with Sal or Ros (Ovx-Sed-Sal; Ovx-Sed-Ros), Ovx trained treated with Sal or Ros (Ovx-Tr-Sal; Ovx-Tr-Ros). Ovx-Sed-Sal rats depicted higher (*P* < 0.05) body weight, plasma total cholesterol (TC) and LDL-C, and liver TC content compared to Sham-Sed-Sal rats. In contrast, mRNA levels of liver PCSK9, LDLR, LRP-1 as well as plasma PCSK9 concentrations and protein levels of LRP-1 were reduced *(P* < 0.01) in Ovx-Sed-Sal compared to Sham-Sed-Sal rats. However, protein levels of LDLR increased *(P* < 0.05) in Ovx-Sed-Sal compared to Sham-Sed-Sal rats. Treatment of Ovx rats with Ros increased (*P* < 0.05) mRNA and protein levels of LRP-1 and PCSK9 but not mRNA levels of LDLR, while its protein abundance was reduced at the level of Sham rats. As a result, plasma LDL-C was not reduced. Exercise alone did not affect the expression of any of these markers in Ovx rats. Overall, Ros treatment corrected Ovx-induced decrease in gene expression of markers of cholesterol metabolism in liver of Ovx rats, but without reducing plasma LDL-C concentrations. Increased plasma PCSK9 levels could be responsible for the reduction of liver LDLR protein abundance and the absence of reduction of plasma LDL-C after Ros treatment.

## Introduction

Incidence of cardiovascular diseases increases with age in women, with a noticeable increase after menopause [1]. Accordingly, menopause as well as ovariectomy (Ovx) in animals is associated with higher plasma levels of low-density lipoprotein cholesterol (LDL-C) and total cholesterol (TC) [2–4]. We previously reported that high plasma levels of LDL-C and TC in Ovx rats were accompanied by a reduction of hepatic LDLR (low-density lipoprotein receptor), PCSK9 (proprotein convertase subtilisin/kexin type 9), SREBP-2 (sterol regulatory element binding protein 2) and LRP-1 (LDL receptor related protein-1) mRNA levels [5]. The importance of estrogens levels in regulating the PCSK9-LDLR axis has also been recently highlighted [6]. This is an important consideration since all of these proteins are involved in circulating cholesterol uptake by the liver and therefore, constitute primary determinants of plasma LDL-C levels [7]. In addition, statins are known to reduce cholesterol synthesis by inhibiting 3-hydroxy-3 methylglutaryl-coenzyme A reductase (HMGCR), the rate limiting enzyme in cholesterol synthesis [8]. The ensuing decrease in liver cholesterol levels leads to the activation of SREBP-2 and up-regulation of hepatic LDLR causing increased clearance of plasma LDL-C [9, 10].

A variety of randomized placebo-controlled trials support the findings that statins, including rosuvastatin (Ros), are effective in reducing plasma LDL-C in hyperecholesterolemic post-menopausal women [11, 12]. However, despite the effectiveness of treatments with Ros and other statins, 50 to 60% of patients fail to reach recommended LDL-C goal defined in the National Cholesterol Education Program Adult Treatment Panel (ATP) III [13–15]. A possible explanation is that statins increase PCSK9 protein levels in a dose-dependent fashion [16, 17]. PCSK9 is secreted by the liver into plasma, binds to the hepatic LDLR at the cell surface and, after endocytosis of the complex, causes its degradation in lysosomes [18, 19]. By decreasing hepatic intracellular levels of cholesterol, via inhibition of *de novo* synthesis, statins increase the activity/nuclear translocation of the transcription factor SREBP-2, which activates both PCSK9 and LDLR gene transcription [17]. The up-regulation of PCSK9 expression [20] blunts the effect of statins by further decreasing LDLR levels [21].

One of the best non-pharmacological strategies for the treatment of hypercholesterolemia due to estrogens deficiency is exercise training [22, 23]. In a cross-sectional study of postmenopausal trained runners, the postmenopausal exercise group showed higher plasma HDL-C and lower LDL-C than the sedentary postmenopausal control group [24]. In Ovx rats, it has been found that exercise training reduced plasma LDL-C and TC [25, 26]. Moreover, the addition of statins to exercise training seems to have potent effects in human. In hypercholesterolemic physically inactive men and women (45–65 years old and postmenopausal), the combination of Ros (10 mg/day) and endurance training reduced plasma LDL-C with a further decrease in oxidized LDL [27]. In a study in which 28 coronary artery disease patients (20 to 89 years old regardless sex) were treated with a combination of Ros (2.5 to 20 mg/day) and aerobic exercise for 20 weeks, a decrease in plasma LDL-C and TC together with an additional increase in plasma HDL-C levels was observed [28]. Although exercise training seems to complement statins treatment, there is no molecular information on how this action may take place. Moreover, considering the previous finding that PCSK9 and HMGCR mRNA gene expression are reduced in Ovx rats [29, 30], it is of interest to gain information on how statins treatment modulates not only LDLR but also PCSK9 and SREBP-2 gene expression in absence of estrogens.

The aim of the present study was to gain hepatic molecular information on the effect of Ros treatment in hypercholesterolemic Ovx sedentary or voluntary trained rats. We targeted regulatory key molecules of hepatic cholesterol metabolism that statins are likely to affect such as LDLR, PCSK9, SREBP-2, LRP-1 (responsible for clearance of circulating lipoprotein remnants) and HMGCR, but also IDOL (inducible degrader of the LDLR) [31] an E3 ubiquitin ligase that induces the degradation of the LDLR in lysosomes, SR-B1 (scavenger receptor class B member 1) [32] involved in cholesterol uptake from high density lipoproteins (HDL), and ACAT-2 (acetyl-CoA acetyltransferase-2) [33] involved in cholesterol esterification.

## Materials and Methods

### Animals

Eight weeks old female Sprague-Dawley strain rats (*n* = 53; Charles River, St Constant, PQ, Canada), weighing 176–189 g upon their arrival were housed individually and had *ad libitum* access to food (chow diet, 12·5% lipid, 63·2% CHO (carbohydrates) and 24·3% protein; kJ from Agribrands Canada, Woodstock, Ontario, Canada) and tap water. Their environment was controlled in terms of light (12 h light–dark cycle starting at 06:00 AM), humidity and room temperature (20–23°C). All experiments described in this report were conducted according to the directives of the Canadian Council on Animal Care after institutional approval Comité de déontologie de l’expérimentation sur les animaux (CDEA).

### Animal Treatment

Seven days after their arrival, rats were either ovariectomized (Ovx, n = 36) or Sham operated (Sham, n = 17). In Ovx rats, both ovaries were excised under isoflurane anesthesia, according to the technique described by Robertson *et al* [34]. Animals were injected with antibiotics (Tribrissen 24%; 0.125 cc/kg, subcutaneously) immediately after surgery. Details of the surgery have been previously described [35]. All rats were, thereafter, kept without any interventions for eight weeks in order to develop metabolic disorders leading to hypercholesterolemia. During this preparatory period, body weight and food intake were measured weekly (S1A Fig).

At the end of the preparatory period (T0 in S1A Fig), all rats were transferred in running wheel cages for three weeks. Sedentary (Sed) rats were housed in cages with locked running wheels. Exercising (Tr) rats were housed in similar cages equipped with unlocked running wheels connected to mechanical switches that recorded revolutions. In addition, sedentary and trained rats were treated either with rosuvastatin (Ros) calcium Crestor AstraZeneca 5mg (5mg/kg of body weight per day, sc) or saline (vehicle; Sal) for three weeks. Among statins, Ros has been reported to be the most effective to lower plasma LDL-C and TC and to increase plasma HDL-C both *in vitro* and *in vivo* [13, 28, 36]. Ros even at dose of 5 mg produces favourable effects on the lipid profile and helps more patients including postmenopausal women to achieve LDL-C goals than comparative statins [37]. Thus, because it had a positive effect in hypercholesterolemic postmenopausal women [11, 38], Ros was chosen for our study.

Sham and Ovx groups were randomly subdivided into subgroups as follows: Sham-Sed-Sal (n = 8), Sham-Sed-Ros (n = 9), Ovx-Sed-Sal (n = 8), Ovx-Sed-Ros (n = 8), Ovx-Tr-Sal (n = 10), and Ovx-Tr-Ros (n = 10) (S1A Fig).

### Sacrifice

At the end of the 3-weeks treatment period (T1 in S1A Fig), rats were euthanized between 08:00 and 12:00 AM. Food was removed from cages overnight before sacrifice. Immediately after complete anaesthesia with isoflurane, the abdominal cavity was opened following the median line of the abdomen and approximately 5mL of blood was collected from the abdominal vena cava with syringes and put into tubes with EDTA (ethylenediaminetetraacetic acid 10.8mg ref 367863). Blood was centrifuged (3000 rpm; 4°C; 10 min; Beckman GPR Centrifuge) and the plasma kept for further analyses. The liver median lobe was freeze-clamped and used for cholesterol, mRNA and proteins determinations. All rats were visually inspected for presence or not of ovaries and uterus were excised and weighed to confirm ovariectomy. Thereafter, all tissue and plasma samples were stored at −78°C until analyses were performed.

### Biochemical Analysis

Plasma triacylglycerol (TAG) concentrations were determined with an enzymatic colorimetric assay available from Sigma (Saint Louis, Missouri, USA). The method described by Folch [39] was used to determine liver cholesterol content. Briefly, 0.1g of liver was homogenized with chloroform–methanol mixture (2:1, v/v). The chloroform layer was collected and evaporated overnight. After adding 10% Triton X-100 in isopropanol, the sample was assayed for total cholesterol using commercial kits (T-cholesterol E cat. 439–17501) according to the manufacturer’s instructions (Wako Diagnostics and Chemicals USA, Richmond, VA, USA). Plasma total cholesterol and HDL-cholesterol levels were determined using Wako kits T- cholesterol E cat.439-17501 and HDL-cholesterol E cat.431-52501, respectively. Plasma LDL-cholesterol level were obtained using Friedewald’s formula (LDL-cholesterol = TC—(TAG/5)–HDL-C) [40]. Plasma PCSK9 concentrations were measured using Mouse/Rat PCSK9 ELISA kit from CircuLex (Cat# CY-8078).

### RNA Isolation and Quantitative Real-time (RT) Polymerase Chain Reaction (PCR)

#### RNA Extraction and cDNA Preparation

Quick-frozen tissue samples of liver were powdered with cold mortar and pestle, and ~100 mg was used for the isolation of RNA. Total RNA was extracted by the guanidine thiocyanate method and mRNA purified using PureLink RNA Mini Kits (Invitrogen) according to the manufacturer’s instruction. Total RNA was reverse transcribed in a final volume of 20 μL using high capacity cDNA reverse transcription kits with random primers (Applied Biosystems, Foster City, CA) as described by the manufacturer. Reverse transcribed samples were stored at -20°C.

#### qPCR Reactions- Taqman® Gene Expression Assays–Endogenous Controls

Gene expression level for endogenous controls was determined using pre-validated Taqman Gene Expression Assays (Applied Biosystems). qPCR reactions for 384 well plate formats were performed using 2 μl of cDNA samples (5-25ng), 5μl of the Fast Universal qPCR MasterMix (Applied Biosystems), 0.5 μl of the TaqMan Gene Expression Assay (20x) and 2.5 μl of water in a total volume of 10 μl. The following assays were used as endogenous controls: HPRT1 (Rn01527840) and b-Actin (Rn00667869).

#### qPCR Reactions- Universal Probe Library (UPL) Assays

Gene expression level for target genes was determined using assays designed with the Universal Probe Library from Roche. qPCR reactions for 384 well plate formats were performed using 2 μl of cDNA samples (5-25ng), 5 μl of the Fast Universal qPCR MasterMix (Applied Biosystems), 2 μM of each primer and 1 μM of a UPL probe in a total volume of 10 μl. The primer sets and probe numbers used to generate amplicons are presented in Table 1.

**Table 1:** Oligonucleotide primers used for quantitative real-time polymerase chain reaction.

**Genes**	**UPL Probe**	**Oligo FWD**	**Oligo REV**
ACAT-2	105	cctcacagatgcgtttcaca	ctctgctcacttgccattttt
b-Actin	17	cccgcgagtacaaccttct	cgtcatccatggcgaact
IDOL	26	ggccatactgtgtgctgtga	atgttccacacgtgatctgc
HMGCR	80	caaccttctacctcagcaagc	acagtgccacacacaattcg
LDLR	16	tgctactggccaaggacat	ctgggtggtcggtacagtg
LRP-1	81	aatcgagggcaagatgacac	ccagtctgtccagtacatccac
PCSK9	89	cacctagcaggtgtggtcag	gcagactgtgcagactggtg
SR-B1	71	ggtgcccatcatttaccaac	gcgagccctttttactacca
SREBP-2	62	gtgcagacagtcgctacacc	aatctgaggctgaaccagga
HPRT1	95	gaccggttctgtcatgtcg	acctggttcatcatcactaatcac

ACAT-2, acyl-CoA cholesterol acyltransferase 2; b-Actin, beta-Actin; IDOL (MYLIP), myosin regulatory light chain interacting protein; HMGCR, 3-hydroxy-3-methylglutaryl-CoA reductase; LDLR, low density lipoprotein receptor; LRP-1, low density lipoprotein receptor-related protein 1; PCSK9, proprotein convertase subtilisin/kexin type 9; SR-B1, scavenger receptor class B, member 1; SREBP-2, sterol regulatory element binding transcription factor2; HPRT1, hypoxanthine phosphoribosyltransferase 1.

#### Detection and Analysis

The ABI PRISM® 7900HT Sequence Detection System (Applied Biosystems) was used to detect the amplification level and was programmed with an initial Step of 3 min at 95°C, followed by 40 cycles of: 5 s at 95°C and 30 s at 60°C. All reactions were run in triplicate and the average values of Cts were used for quantification. HPRT1 and b-Actin were used as endogenous controls. The relative quantification of target genes was determined using the ΔΔCT method. Briefly, the Ct (threshold cycle) values of target genes were normalized to an average Ct of HPRT1 and b-Actin. (ΔCT = Ct_target_—Ct_endo_) and compared with a calibrator: ΔΔCT = ΔCt_Sample_—ΔCt_Calibrator_. Relative expression (RQ) was calculated using the formula is RQ = 2^-ΔΔCT^ with the help of SDS2.2.2 and Data Assist 3.0 software (Applied Biosystems). The ΔCt standard deviation (ΔCtSD) was used to test quality of technical triplicates for the gene of interest and the endogenous gene for the same sample. The standard deviations for column charts were calculated with final 2^-ΔΔCT^ values of each group.

### Western Blot Analysis

Livers were frozen and pulverized under liquid nitrogen with a pre-chilled mortar and pestle. Approximately 50-100mg of liver powders of each rat were homogenized in ice-cold RIPA buffer (50mM Tris, 150 mM NaCl, 1 mM EDTA, 1% Triton X-100, 0.5% sodium deoxycholate, 0.1% SDS, pH 7.4) containing protease inhibitor cocktail (Roche). Liver homogenates were shaken for 30 min at 4°C before centrifugation at 12000 rpm for 15 min. Proteins of the resulting supernatants were quantified using BCA protein assay reagent. An equal amount of homogenate proteins from liver samples were separated by 8% SDS-polyacrylamide gel electrophoresis, blotted on nitrocellulose membranes (Bio-Rad), and blocked for 1 h in Tris-buffered saline-Tween 20 (TBS-T; 50 mm Tris-HCl, pH 7.5, 150 mm NaCl, 0.1% Tween 20) containing 5% nonfat dry milk. Membranes were then incubated overnight in TBS-T supplemented with 1% nonfat milk and the indicated antibodies: goat anti-mouse LDLR (1:1000; catalog no: AF2148 or A2255, R&D Systems), rabbit anti-mouse/rat LRP-1 (1:10 000; catalog no: L2170 Sigma-Aldrich) and rabbit anti-β-actin (1:2500; catalog no: A2066, Sigma-Aldrich). Appropriate HRP-conjugated secondary antibodies (1:10 000; GE healthcare) were used for detection using the Western Lightning Ultra chemiluminescence kit (catalog no. NEl112001EA, PerkinElmer Life Sciences) and BioFlex EC Films (catalog no. CLEC810, InterScience).

### Statistical Analysis

All data are expressed as means ± SD. Due to the design of the study, two statistical analyses were conducted. To establish statistical significance between Ovx groups, a two-way analysis of variance (ANOVA) for non-repeated measures was applied, using rosuvastatin treatment and voluntary training as main effects. Fisher’s *post-hoc* test was used in the event of a significant (*p* < 0.05) *F* ratio. One-way ANOVA followed by Fisher’s *post-hoc* test was used to detect differences (*p* < 0.05) between Sham-Sed-Sal group and all Ovx groups. Student’s t test was used to detect differences between Sham-Sed-Sal and Sham-Sed-Ros groups. The robustness of experimental design of 2x2 with 8 to 10 rats/group brings confidence to the assumption of equality of variances. The SD of the data used for the two-way AVOVA did not vary more than a factor from 1 to 2 which speaks in favour of the homogeneity.

## Results

### Body Weight and Uterus Weight

Body weight measured at the end of the experiment (T1, S1A Fig) significantly (*p* ˂ 0.01) increased in Ovx compared to Sham rats (Table 2). There was no difference in body weight between all Ovx animals or between Sham rats (S1B Fig). Food intake was significantly (*p* ˂ 0.01) higher in both Ovx Tr groups of animals independently of the Ros treatment. Ovx rats showed significantly (*p* ˂ 0.001) lower uterine wet weights compared to Sham rats confirming the efficiency of the surgery (Table 2).

**Table 2:** Anthropometric, plasma and liver metabolic variables.

**Variables**	**Sham-Sed-Sal**	**Sham-Sed-Ros**	**Ovx-Sed-Sal**	**Ovx-Sed-Ros**	**Ovx-Tr-Sal**	**Ovx-Tr-Ros**
Body weight (g)	344 ± 8	347 ± 16	417 ± 14**	407 ± 11**	407 ± 10**	406 ± 8**
Food intake (g/wk)	23 ± 0.6	21 ± 1.6	25 ± 0.4	25 ± 0.7	31 ± 1.3 **^&&^	30 ± 0.6**^&&^
Uterus weight (mg)	0.57 ± 0.03	0.68 ± 0.04	0.12 ± 0.01***	0.12 ± 0.01***	0.12 ± 0.01***	0.12 ± 0.01***
Plasma TAG (g/L)	0.55 ± 0.06	0.62 ± 0.11	0.63 ± 0.07	0.48 ± 0.06	0.43 ± 0.03^&^	0.38 ± 0.04^&^
Plasma TC (mmol/L)	1.69 ± 0.05	1.90 ± 0.11	2.16 ± 0.16*	2.23 ± 0.14*	2.17 ± 0.1*	2.03 ± 0.14*
LDL-cholesterol (mmol/L)	0.56 ± 0.05	0.83 ± 0.04	0.95 ± 0.2*	1.2± 0.15*	0.95 ± 0.08*	1.11 ± 0.11*
HDL-cholesterol (mmol/L)	1.02 ± 0.06	0.95 ± 0.07	1.08 ± 0.06	0.90 ± 0.05^§^	1.14 ± 0.07	0.84 ± 0.05^§^
TC/HDL-C	1.7 ± 0.09	2.03 ± 0.05^§^	2.03 ± 0.17*	2.51 ± 0.21*^§^	1.95 ± 0.09	2.46 ± 0.12*^§^
LDL-C/HDL-C	0.60 ± 0.08	0.90 ± 0.05^§^	0.91 ± 0.2*	1.41 ± 0.2*^§^	0.87 ± 0.08	1.37 ± 13*^§^
Liver TC (mg/g)	13.2 ± 0.5	11.6 ± 0.3	14.2 ± 1.02*	14.2 ± 0.53*	15.9 ± 1.12*	15.2 ± 1.2*

Values are mean ± SD with n = 8–10 rats per group.

* *P* < 0.05

** *P* < 0.01

*** *P* < 0.001 significantly different from Sham-Sed-Sal.

^&^ *P* < 0.05

^&&^ *P* < 0.01; significantly different from sedentary (Sed) counterparts.

^§^ *P* < 0.05; significantly different from saline (Sal) counterparts. TAG: triacylglycerol; TC: total cholesterol; LDL: low density lipoprotein; HDL: high density lipoprotein. LDL-cholesterol = TC—(TAG/5)–HDL-C (Friedewald’s formula).

### Plasma and Liver Lipid Profile

Plasma TAG did not undergo any significant changes in Ovx Sed rats treated or untreated with Ros compared to Sham rats (Table 2). In contrast, plasma TAG concentrations were significantly (*p* ˂ 0.05) lower in Ovx rats submitted to voluntary training alone or in combination with Ros, suggesting a positive effect of training on lipid metabolism. Ovariectomy led to significant (*p* ˂ 0.05) increases in plasma TC, LDL-C and liver TC levels compared to Sham-Sed-Sal rats. Treatments of Ovx animals with both Ros and voluntary training alone or in combination were not able to correct these increases. Plasma HDL-C concentration was not changed in Ovx compared to Sham rats. On the opposite, treatment with Ros significantly decreased (*p* ˂ 0.05) plasma HDL-C concentrations both in Ovx sedentary and trained rats compared to their Sal counterparts. Voluntary training alone did not change HDL-C in Ovx rats. Atherogenic indexes, TC/HDL-C and LDL/HDL-C ratios were significantly (*p* < 0.05) increased by Ros treatment alone or combined with exercise both in Sham and Ovx rats, while voluntary training alone had no effect (Table 2). The average of daily running distance (S1C Fig.) was 1.67 ± 0.16 km for Ovx rats treated with saline and 1.54 ± 0.2 km for Ovx rats treated with Ros.

### Hepatic Gene Expression

**Fig 1:**
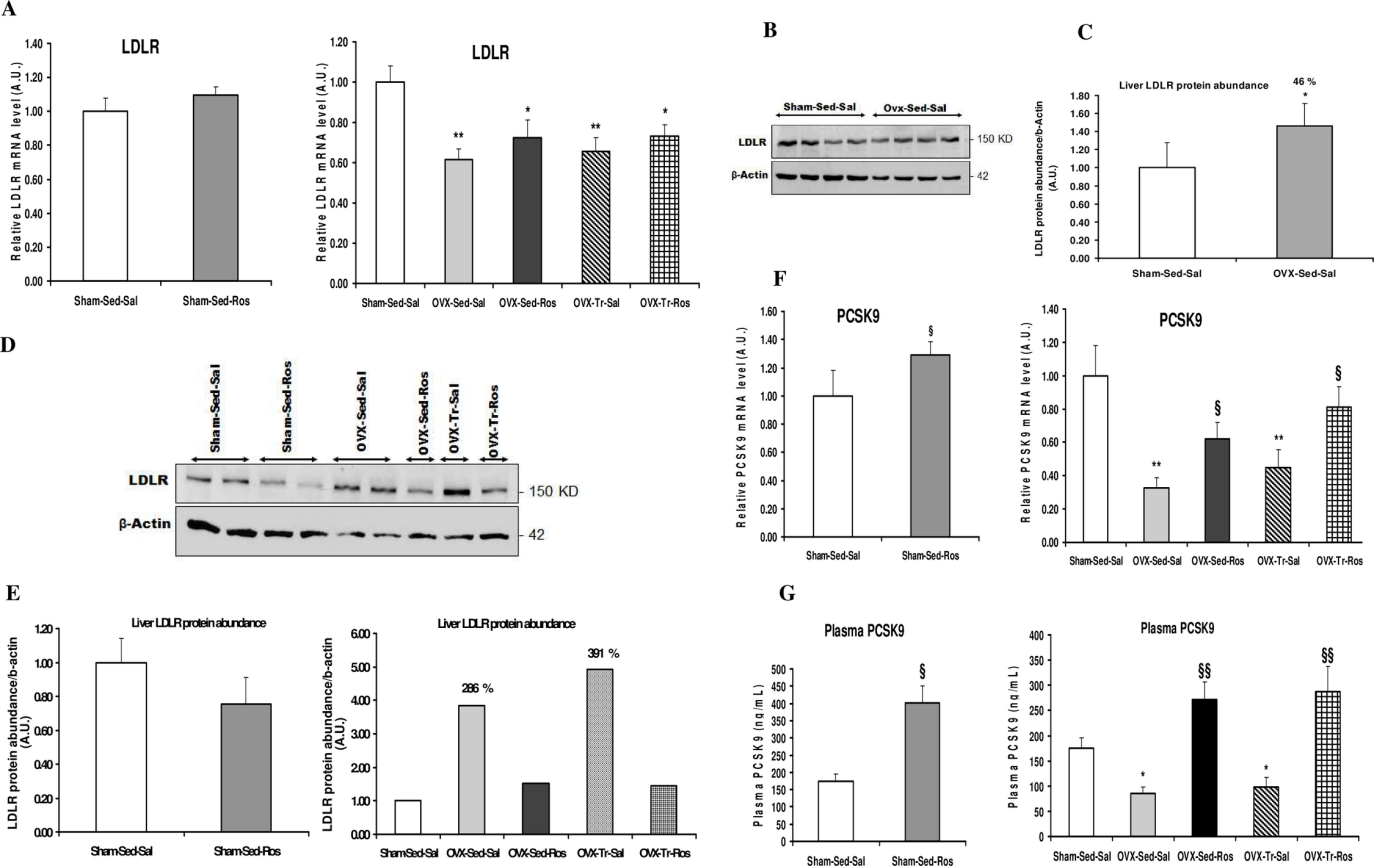
Liver gene expression of LDLR and PCSK9 in ovariectomized rats treated with rosuvastatin. Liver mRNA (**A**) and protein expression (**D, E**) of low-density lipoprotein receptor (LDLR) are, respectively, reduced and increased in ovariectomized (Ovx) rats either in sedentary (Sed) and trained (Tr) states without significant effects of rosuvastatin (Ros) treatment, whereas hepatic mRNA (**F**) and plasma levels (**G**) of proprotein convertase subtilisin/kexin type 9 (PCSK9) were decreased in Ovx rats either in Sed and Tr states and increased with Ros treatment. Values for mRNA as determined by quantitative polymerase chain reaction are expressed as a ratio of the control value (1.0) and are mean ± SD (standard deviation) with n = 8–10 rats per group. Hprt1 (hypoxanthine phosphoribosyltransferase 1) and b-actin were used as endogenous controls. *p < 0.05; ** *p* < 0.01 significantly different from Sham-Sed-Sal. ^§^*p* < 0.05; ^§§^*p* < 0.01 significantly different from respective Saline (Sal) group. Protein abundance in liver of rats were determined by Western blot analysis and normalized to those of b-actin. Quantification (**C**) of proteins bands in (**B**) with n = 4 rats/group. Quantification (**E**) of proteins bands in (**D**) with n = 1 to 2 rats/group. The % changes indicated (E) are in comparison to the Sham-Sed-Sal group.

Bilateral Ovx resulted in an approximately 40% (*p* < 0.01) reduction in liver LDLR mRNA levels when compared to Sham-Sed-Sal animals (Fig 1A). However, protein levels of liver LDLR increased in Ovx-Sed-Sal compared to Sham-Sed-Sal (Fig 1B and 1C). Treatment with Ros alone or combined to voluntary exercise tended to increased LDLR mRNA levels in Ovx rats by ~10% as compared to the Sal counterparts but did not reach the significance level (*p* = 0.25) (Fig 1A). Liver LDLR protein levels were reduced at the level of Sham rats in Ovx rats after treatment with Ros (Fig 1D and 1E). Moreover, protein abundance in liver of Sham rats (D and E) after Ros treatment (Sham-Sed-Ros) showed a tendency to decrease as compared to Sal counterparts. On the other hand, PCSK9 mRNA expression as well as plasma levels of PCSK9 were decreased (*p* < 0.05) in Ovx compared to Sham rats in the Sed-Sal condition (Fig 1F and 1G). Ros treatment significantly reversed these decreases in Ovx rats when used alone (*p* < 0.05) and when combined with voluntary training (*p* < 0.05). Exercise training alone or in combination with Ros did not affect LDLR and PCSK9 responses in Ovx rats.

**Fig 2:**
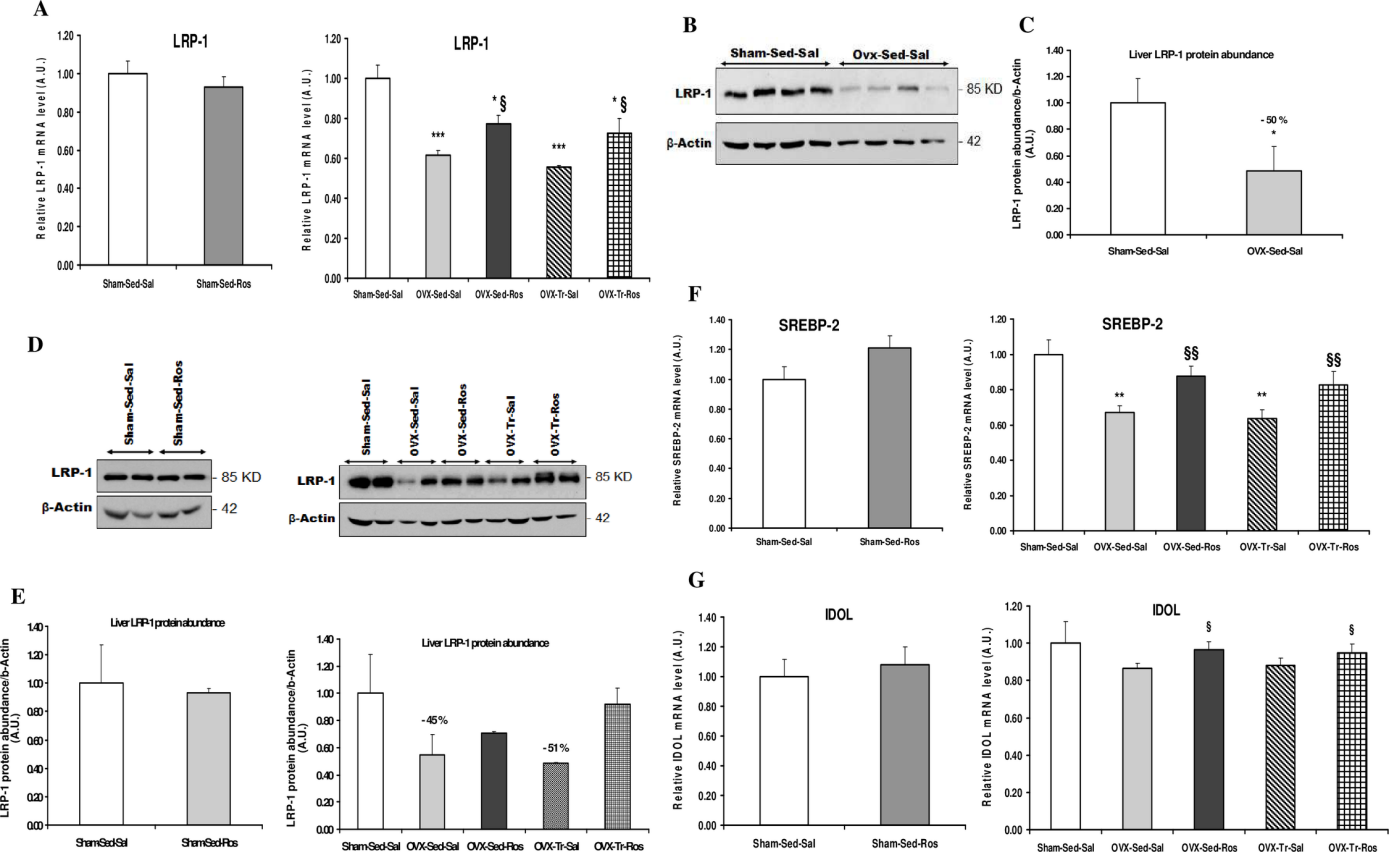
Liver gene expression of LRP-1, SREBP-2 and IDOL in ovariectomized rats treated with rosuvastatin. Liver mRNA (**A**) and protein abundance (**B** and **D**) of low-density lipoprotein receptor-related protein 1 (LRP-1) along with mRNA expression (**F**) of sterol regulatory element binding protein 2 (SREBP-2) were reduced in ovariectomized (Ovx) rats either in sedentary (Sed) and trained (Tr) states and increased with rosuvastatin (Ros) treatment, whereas liver mRNA (**G**) of inducible degrader of the LDLR (IDOL) was not changed in Ovx rats but was increased by Ros treatment. Values for mRNA as determined by quantitative polymerase chain reaction are expressed as a ratio of the control value (1.0) and are mean ± SD (standard deviation) with n = 8–10 rats per group. Hprt1 (hypoxanthine phosphoribosyltransferase 1) and b-actin were used as endogenous controls. *p < 0.05; ** *p* < 0.01; *** *p* < 0.001 significantly different from Sham-Sed-Sal. ^§^*p* < 0.05; ^§§^*p* < 0.01 significantly different from respective Saline (Sal) group. Protein abundance in liver of rats were determined by Western blot analysis and normalized to those of b-actin. Quantification (**C**) of proteins bands in (**B**) with n = 4 rats/group. Quantification (**E**) of proteins bands in (**D**) with n = 2 rats/group. The % changes indicated (E) are in comparison to the Sham-Sed-Sal group.

LRP-1 mRNA and protein levels were lower in Ovx compared to Sham rats and increased with Ros treatment in Ovx rats without any effects of exercise training (Fig 2A–2E). The same pattern of response was observed for SREBP-2 (Fig 2F). IDOL gene expression only showed a tendency to be decreased in Ovx rats but was significantly (*p* < 0.05) increased with Ros treatment without any effect of exercise training (Fig 2G).

**Fig 3:**
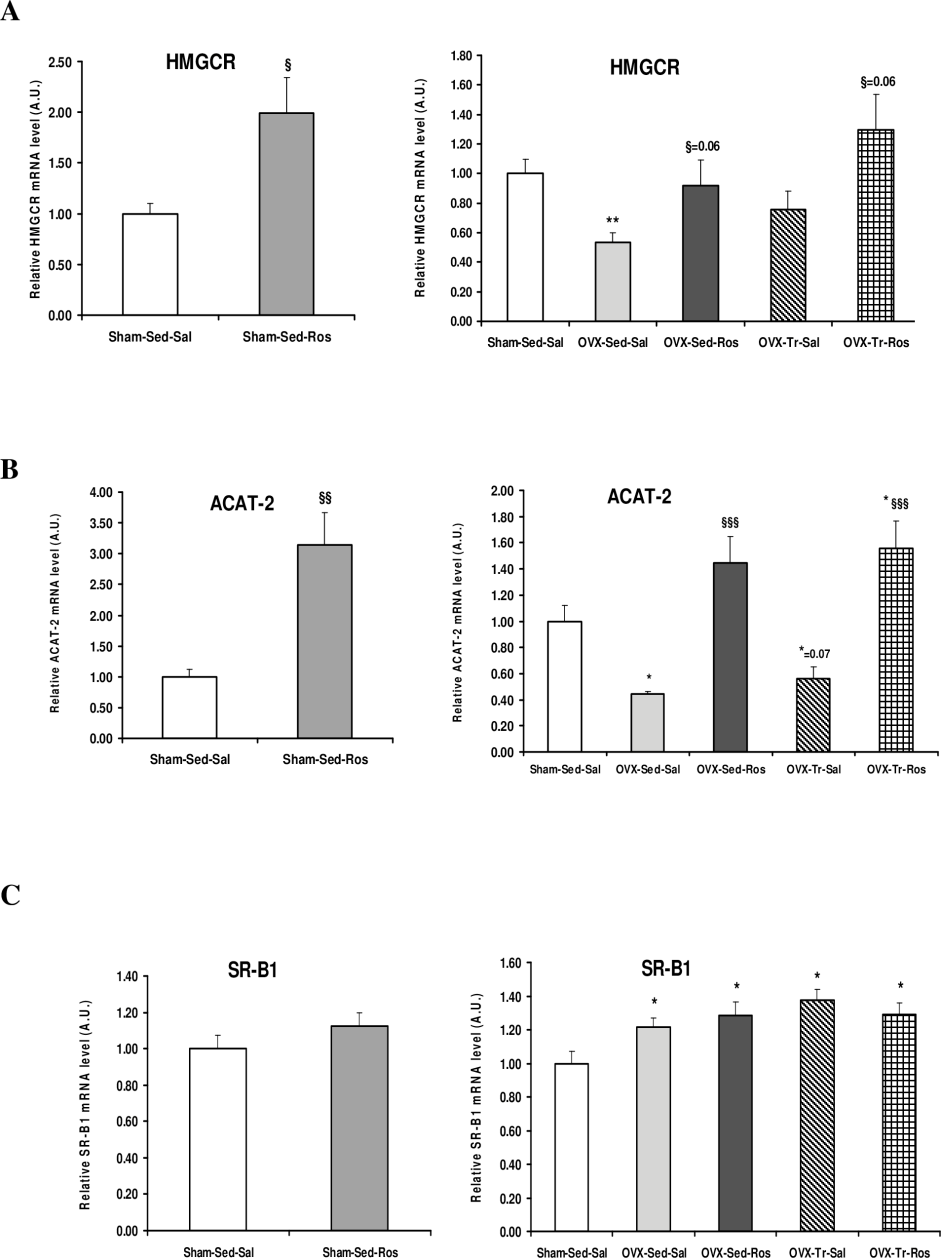
Liver mRNA expression of HMGCR, ACAT-2 and SR-B1 in ovariectomized rats treated with rosuvastatin. Liver mRNA expressions of enzymes 3-hydroxy-3-methyl-glutaryl-CoA reductase (HMGCR) (**A**) and acetyl-CoA acetyltransferase-2 (ACAT-2) (**B**) involved in cholesterol synthesis and esterification, respectively, are decreased in ovariectomized (Ovx) rats in sedentary (Sed) and trained (Tr) states and increased with rosuvastatin (Ros) treatment while transcripts of cholesterol transporter scavenger receptor class B member 1 (SR-B1) (**C**) were increased in Ovx animals without any effect of Ros. Values for mRNA as determined by quantitative polymerase chain reaction are expressed as a ratio of the control value (1.0) and are mean ± SD (standard deviation) with n = 8–10 rats per group. Hprt1 (hypoxanthine phosphoribosyltransferase 1) and b-actin are used as endogenous controls. * Significantly different from Sham-Sed-Sal, *p* < 0.05; ** *p* < 0.01; *** *p* < 0.001. ^§^ Significantly different from respective Saline (Sal), *p* < 0.05; ^§§^*p* < 0.01; ^§§§^*p* < 0.001.

HMGCR gene expression was reduced (*p* < 0.05) in Ovx-Sed-Sal compared to Sham-Sed-Sal animals and increased (*p* < 0.06) by the Ros treatment in both Sed and Tr rats (Fig 3A). The same pattern of Ovx-induced decrease and Ros-induced increase in Sed and Tr animals was found for ACAT-2 that encodes the enzyme responsible for intracellular esterification of cholesterol (Fig 3B). On the opposite, mRNA levels of liver SR-B1, a receptor that allows the hepatic uptake of cholesteryl ester from HDL, was significantly (*p* ˂ 0.05) increased in all Ovx compared to Sham rats (Fig 3C). This increase was neither affected by Ros treatment or voluntary training alone, nor by the combination of both of them. The only significant effect of Ros in Sham animals was an increase in mRNA levels for HMGCR, ACAT-2, and PCSK9 (Figs 1 and 3).

## Discussion

Results of the present study first confirm the recent findings that bilateral Ovx in rats causes a reduction in mRNA levels of key proteins involved in cholesterol metabolism including LDLR, PCSK9, LRP1, SREBP-2 and HMGCR, which is correlated with an increase in plasma and liver cholesterol levels [5]. Considering these alterations in Ovx rats, the present study addressed the concern of the action of statins treatment in this animal model.

The main finding of the present study was that three weeks of Ros treatment alone or in combination with voluntary exercise in Ovx rats resulted in a significant attenuation of the Ovx-induced reduction in mRNA expression of PCSK9, LRP-1 and SREBP-2. However this attenuation did not reach statistical significance (p = 0.25) for LDLR mRNA levels. The PCSK9 and LRP-1 response to Ovx and Ros was also confirmed at the protein level. Paradoxically, the abundance of LDLR protein increased in liver of Ovx rats and was reduced to the level of Sham rats following Ros treatment. This was closely associated with absence of reduction of plasma LDL-C levels and atherogenic indexes after Ros administration in Ovx rats. Overall, these results indicate that Ros treatment induced an up-regulation of cholesterol metabolism gene transcription in the rat liver that was reduced by estrogens deficiency. However, the question is why this up-regulation of gene transcription following Ros treatment was not able to reduce hypercholesterolemia in Ovx rats.

It is well known that statins treatment induce SREBP-2 activation and translocation to the nucleus where it activates LDLR and PCSK9 genes transcription through direct interaction with the sterol-regulatory element 1 (Sre-1) in their promoters [17, 41]. PCSK9 expression is primarily regulated at the transcription level, and PCSK9 mRNA abundance correlates with its protein expression [41]. Large amounts of PCSK9 are synthetized and secreted by the liver before interacting with transmembrane proteins [42–44]. Our data showed that Ros-induced increase in SREBP-2 mRNA levels in Ovx rats was matched with increases in both mRNA and plasma PCSK9 levels. However, the same pattern of response was not observed with mRNA and protein levels of LDLR. While mRNA levels of LDLR slightly increased (not significant) with Ros treatment, its protein abundance was reduced at the level of Sham in liver of Ovx rats and, as a consequence, plasma LDL-C levels were not reduced. The LDLR activity is regulated at both transcriptional and post-translational levels. The post-translational regulation of LDLR is mediated by PCSK9 that can bind LDLR protein, intracellularly [45] and extracellularly [18, 43] and direct the complex to lysosome for degradation. At transcriptional level, LDLR is regulated by SREBP-2 that binds to Sre-1 in the promoter region of LDLR gene and up-regulates its transcription that ultimately increases clearance of LDL from the bloodstream [17]. However, this transcriptional regulation of the LDLR is paradoxical because SREBP-2 also increases the transcription of PCSK9 that in turn accelerates LDLR protein degradation in the liver, thereby limiting uptake of plasma LDL particles. Thus, two opposing effects on plasma cholesterol levels are initiated by the same metabolic signal. Consequently, the significant induction of PCSK9, which functionally mediates LDLR protein degradation, could be a possible explanation for the reduction of liver LDLR protein and the absence of lower circulating LDL-C levels in Ovx rats after Ros treatment. Although scarce, there are studies supporting the absence of effects of statins in lowering LDL-C in rodents. In a study reporting data on liver LDLR in Ovx animals following statins treatment, it was found that simvastatin had no effects on liver LDLR mRNA expression in Ovx rabbits fed cholesterol diet, 12 weeks after treatment [46]. In Ovx golden Syrian hamsters fed hypercholesterolemic diet, simvastatin (10 mg/kg) failed to decrease plasma LDL-C when treated for 12 weeks and when treated 4 weeks after being Ovx for 8 weeks [47].

A significant increase in liver mRNA and plasma levels of PCSK9 was observed in Sham sedentary rats after Ros treatment. However, Ros treatment in Sham animals did not affect mRNA levels of SREBP-2 and LDLR and protein abundance of LDLR. This suggests that in addition to SREBP-2 another factor could be involved in the induction of PCSK9 during treatment with Ros in rats. Dong et al [48] reported that Ros treatment in hamsters, not only increased the expression of SREBP-2 but also stimulated the mRNA and protein expression of hepatocyte nuclear factor (HNF1a), a pivotal transcription activator of the Pcsk9 gene [49]. In this study we also measured mRNA expression of HNF1a, but no induction was observed following Ros treatment both in Sham and Ovx rats (S1D Fig). Therefore the higher induction of PCSK9 expression by Ros treatment in Sham rats remains to be clarified.

In addition to LDLR, SREBP-2 also participates to the regulation of LRP-1 gene expression [50]. LRP-1 binds to apolipoprotein E and serves as a receptor for remnant lipoproteins in the liver, thus playing an important role in clearing these atherogenic particles [51, 52]. Moon et *al* [50] demonstrated that atorvastatin increases LRP-1 and the nuclear form of SREBP-2 in HepG2 cells, and that when SREBP-2 was knocked down by small interfering RNA, the induction of LRP-1 expression by atorvastatin did not take place [50]. These results suggest that SREBP-2 acts as a mediator of statin-induced up-regulation of hepatic LRP-1 gene expression. We recently reported a decrease in liver LRP-1 and SREBP-2 gene expression in 8-weeks Ovx rats [5] that is confirmed in the present study. The present increase in LRP-1 gene expression following Ros treatment in absence of significant effects on LDLR gene expression rises interesting questioning. It has been recently reported that in addition to LDLR, LRP-1 is a PCSK9 target in HepG2 cells but that the degradation machinery is different [53]. These authors raised the possibility that LDLR can effectively compete with LRP-1 for PCSK9 activity. Ros injection by increasing mRNA and protein levels of hepatic LRP-1 in Ovx rats, could contribute to a reduction in circulating lipoproteins remnants, improving in part the plasma atherogenic profile.

To go one step further, we measured gene expression of IDOL an ubiquitin ligase that also mediates the degradation of the LDLR. IDOL is controlled at the transcriptional level by liver X receptor (LXR) independently of the SREBP pathway [54]. IDOL mRNA was not affected by the ovariectomy in the present study as observed by Roubtsova et al [55] in Ovx mice. These authors also reported an absence of effects of estrogens treatment on IDOL transcripts. On the other hand, the present observation of a reduction in hepatic mRNA levels of HMGCR in Ovx rats has been previously reported [29]. Treatment of Ovx rats with Ros increased mRNA levels of this gene in liver. An increase was also observed in Sham rats treated with Ros. Roglans et *al* [56] previously reported that atorvastatin and simvastatin also increased mRNA levels of HMGCR in normolipidemic rats fed a standard diet. However, it is likely that the activity of this enzyme remains inhibited by the action of the drug. If on one hand, Ros treatment in the present study did not lower plasma LDL-C, on the other hand, it did lower plasma HDL-C concentration. It is important to recall that a significant portion of cholesterol in blood is transported by HDL in rats [57]. SR-B1 in liver is the transporter responsible for the uptake of plasma cholesteryl esters from mature HDL. As previously reported, SR-B1 mRNA levels were higher in Ovx than in Sham rats [5]. Ros treatment did not, however, alter gene expression of SR-B1 in Ovx animals making it unlikely that the lower levels of HDL-C in Ros-treated Ovx rats be due to a change in SR-B1 expression.

Similarly to PCSK9 and LRP-1, ACAT-2 mRNA levels were decreased in Ovx compared to Sham rats, this reduction being cancelled out when Ovx animals were treated with Ros. Treatment with Ros also induced ACAT-2 mRNA levels in Sham sedentary rats. ACAT-2 is highly expressed in human and rodent hepatocyte and plays a key role in the hepatic storage and packaging of cholesteryl ester into apoB-containing lipoproteins (VLDL) [33, 58]. Little is known about the regulation of ACAT-2, but it seems that in hepatocytes ACAT-2 is transcriptionally stimulated by HNF1a [58, 59]. However, since the expression of HNF1a remained unchanged (S1D Fig), it seems that another factor could be involved in the induction of ACAT-2 in female rats during Ros treatment. The reduction of mRNA levels of liver ACAT-2 in Ovx rats suggests a reduction in the synthesis and assembly of VLDL. Accordingly, a decrease in VLDL production has been reported in Ovx rats [60]. Does Ros treatment induce an increase in VLDL synthesis and secretion in Ovx animals is a question that remains to be determined.

With regards to the effects of voluntary exercise training alone, we found that plasma and liver cholesterol concentrations were not reduced in Ovx rats after three weeks of exercise. This was associated with an absence of change on gene expression of key molecules involved in cholesterol metabolism. This might be linked to the fact that rats being ovariectomized for 8 weeks before being placed in running wheels increased their body weights that may in turn explain the relatively low intensity of voluntary exercise (~1.6 km/day). The intensity and duration of exercise may have been too weak to stimulate gene expression of gens involved in cholesterol metabolism. In a previous study we found that 8 weeks of treadmill exercise training has no effects on expression of most of the genes involved in hepatic cholesterol metabolism in Sham and Ovx rats [5]. It is possible that exercise training may regulate plasma and hepatic cholesterol levels by different mechanisms such as increased elimination of cholesterol through bile acids excretion [61] but the evidence that it does affect hepatic cholesterol metabolism at the molecular level is lacking.

In summary, results of the present experiments first indicate that several key molecules of cholesterol metabolism (PCSK9, LRP-1, HMGCr, and ACAT-2) are reduced at the transcriptional level in liver of Ovx compared to Sham rats. Rosuvastatin treatment in Ovx rats increased the expression of these genes suggesting that statins may contribute at the molecular level to the proper regulation of hypercholecterolemia with estrogens withdrawal. However, the absence of effects of Ros on plasma cholesterol levels in the present Ovx rats might be linked to the higher induction of PCSK9 that degrades the LDLR protein, decreasing clearance of circulating LDL and remnant lipoproteins.

## Supporting Information

S1 Fig Supplementary figures. **S1A** shows details of experimental protocol. **S1B** exhibits body weight gain during the experiment which is significantly increased in all Ovx groups compared to Sham groups. There was no difference in body weight between Ovx groups or between Sham groups. **S1C,**is running distance. The average of daily running distance was 1.67 ± 0.16 km for ovariectomized (Ovx) rats treated with saline (Sal); and 1.54 ± 0.2 km for Ovx rats treated with rosuvastatin (Ros). The activity of Ovx rats treated with Ros showed a tendency to decrease (not statistically significant) when compared to Ovx rats treated with saline. **S1D** represents liver mRNA expression of HNF1a. Liver mRNA expression of hepatocyte nuclear factor (HNF1a) was not induced following Ros treatment both in Sham and Ovx rats.
(PDF)
