# Supplementary material for: Combined Effects of Rosuvastatin and Exercise on Gene Expression of Key Molecules Involved in Cholesterol Metabolism in Ovariectomized Rats
Source: PLoS One. 2016 Jul 21;11(7):e0159550. doi: 10.1371/journal.pone.0159550 (PMC4956224; doi:10.1371/journal.pone.0159550)
Supplement: S1 Fig — S1A shows details of experimental protocol. S1B exhibits body weight gain during the experiment which is significantly increased in all Ovx groups compared to Sham groups. There was no difference in body weight between Ovx groups or between Sham groups. S1C,is running distance. The average of daily running distance was 1.67 ± 0.16 km for ovariectomized (Ovx) rats treated with saline (Sal); and 1.54 ± 0.2 km for Ovx rats treated with rosuvastatin (Ros). The activity of Ovx rats treated with Ros showed a tendency to decrease (not statistically significant) when compared to Ovx rats treated with saline. S1D represents liver mRNA expression of HNF1a. Liver mRNA expression of hepatocyte nuclear factor (HNF1a) was not induced following Ros treatment both in Sham and Ovx rats. (PDF) [file pone.0159550.s001.pdf]

S1 Fig. Supplementary figures

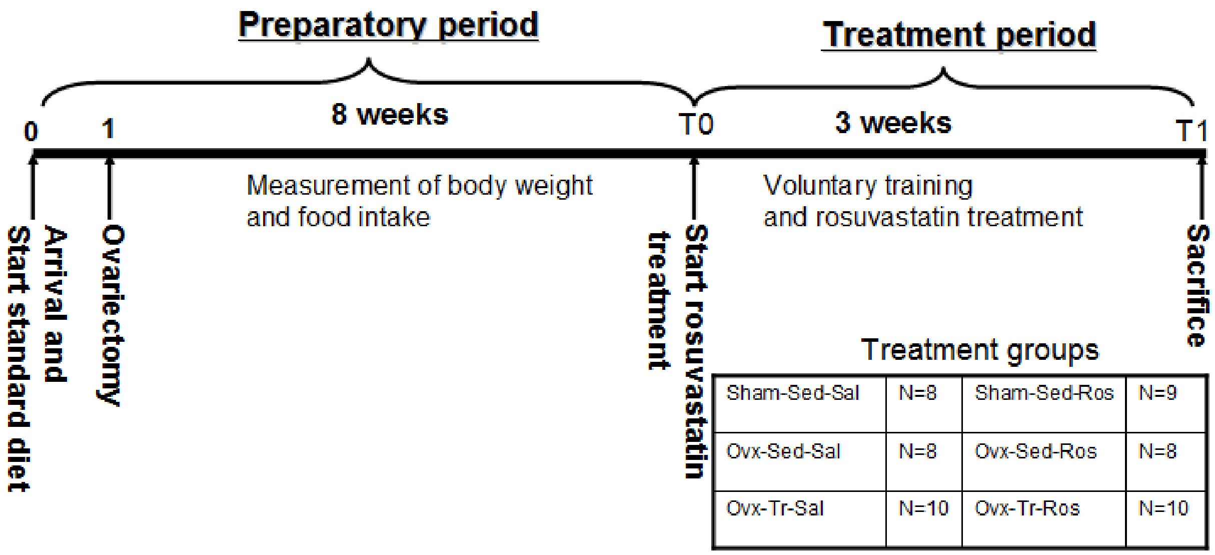

S1A Fig. Experimental protocol. O, arrival of rats in the animal room. **1**, one week after arrival, rats were ovirectomized (Ovx) or Sham operated (Sham). **Preparatory period,** Ovx and Sham rats were kept without any interventions for eight weeks in order to develop metabolic disorders leading to hypercholesterolemia. **T0**, end of the preparatory period, and all rats were transferred in running wheel cages for three weeks of voluntary exercise and rosuvastatin (Ros) treatment. **T1**, end of experiment, all rats were sacrificed. Six groups were formed as follow: **Sham-Sed-Sal**, sham sedentary rats treated with saline; **Sham-Sed-Ros**, sham sedentary rats treated with Ros; **Ovx-Sed-Sal**, Ovx sedentary rats treated with saline **Ovx-Sed-Ros**, Ovx sedentary rats treated with Ros; **Ovx-Tr-Sal**, Ovx trained rats treated with saline; and **Ovx-Tr-Ros**, Ovx trained rats treated with Ros.

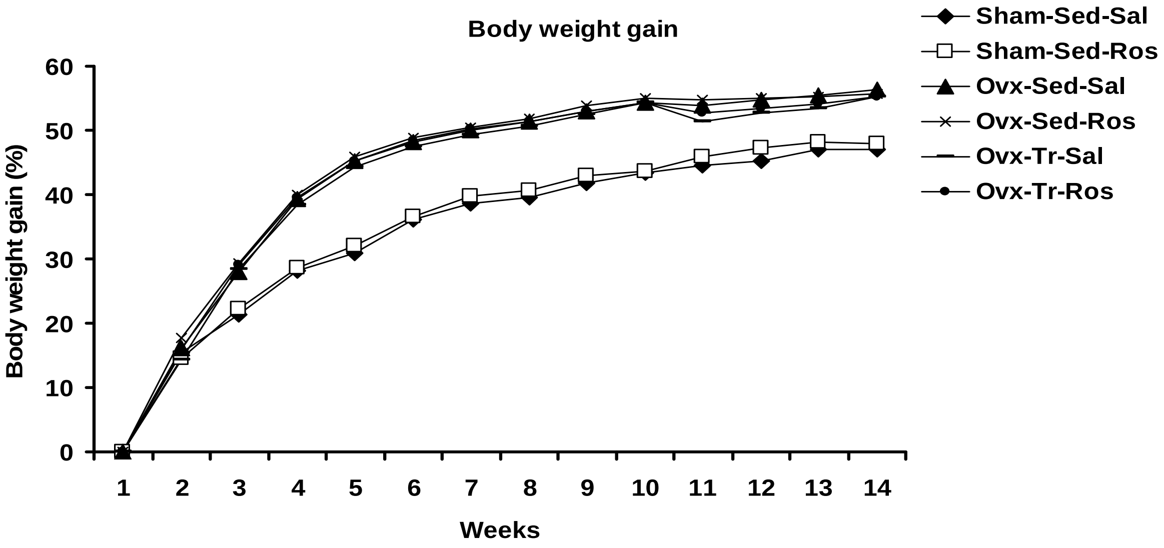

S1B Fig. Body weight gain during the experiment. Body weight significantly increased in all Ovx groups compared to Sham groups. There was no difference in body weight between Ovx groups or between Sham groups. **Sham-Sed-Sal**, sham sedentary rats treated with saline; **Sham-Sed-Ros**, sham sedentary rats treated with Ros; **Ovx-Sed-Sal**, Ovx sedentary rats treated with saline **Ovx-Sed-Ros**, Ovx sedentary rats treated with Ros; **Ovx-Tr-Sal**, Ovx trained rats treated with saline; and **Ovx-Tr-Ros**, Ovx trained rats treated with Ros.

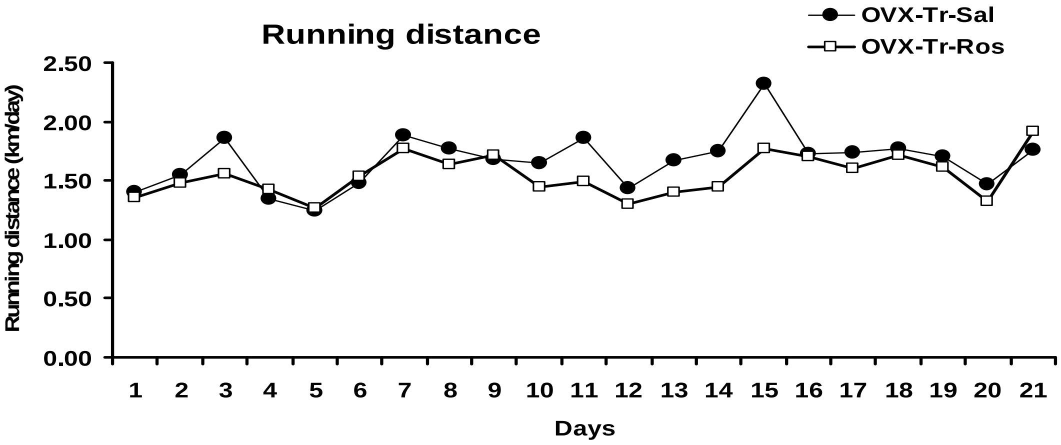

S1C Fig. Running distance. The average of daily running distance was 1.67 ± 0.16 km for ovariectomized (Ovx) rats treated with saline (Sal); and 1.54 ± 0.2 km for Ovx rats treated with rosuvastatin (Ros). The activity of Ovx rats treated with Ros showed a tendency to

decrease (not statistically significant) when compared to Ovx rats treated with saline. **Ovx-Tr-Sal**, Ovx trained rats treated with saline; and **Ovx-Tr-Ros**, Ovx trained rats treated with Ros.

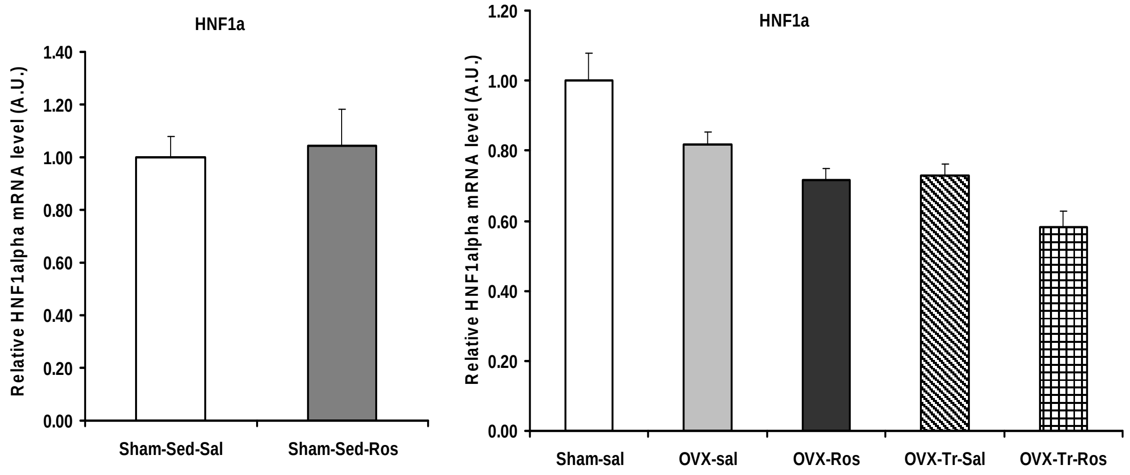

S1D Fig. **Liver mRNA expression of HNF1a**. Liver mRNA expression of hepatocyte nuclear factor (HNF1a) was not induced following Ros treatment both in Sham and Ovx rats.
